# Supplementary material for: Above- and belowground biodiversity jointly tighten the P cycle in agricultural grasslands
Source: Nat Commun. 2021 Jul 21;12:4431. doi: 10.1038/s41467-021-24714-4 (PMC8295381; doi:10.1038/s41467-021-24714-4)
Supplement: Supplementary file 1 — Supplementary Info [file 41467_2021_24714_MOESM1_ESM.pdf]

***Supplementary Information* for**

Above- and belowground biodiversity jointly tighten the P cycle

Yvonne Oelmann, Markus Lange, Sophia Leimer, Christiane Roscher, Felipe Aburto, Fabian Alt, Nina Bange, Doreen Berner, Steffen Boch, Runa S. Boeddinghaus, François Buscot, Sigrid Dassen, Gerlinde De Deyn, Nico Eisenhauer, Gerd Gleixner, Kezia Goldmann, Norbert Hölzel, Malte Jochum, Ellen Kandeler, Valentin H. Klaus, Till Kleinebecker, Gaëtane Le Provost, Peter Manning, Sven Marhan, Daniel Prati, Deborah Schäfer, Ingo Schöning, Marion Schrumpf, Elisabeth Schurig, Cameron Wagg, Tesfaye Wubet, Wolfgang Wilcke

Supplementary Table 1: Correlation (Pearson) matrix of variables in the biodiversity experiment. Significant negative and positive relationships in blue and orange, respectively (hue refers to the closeness of the relationship). Asterisks refer to the level of significance (*** $p < 0.001$, ** $p < 0.01$, * $p < 0.05$). The Benjamini & Hochberg correction was applied for the multiple testing (see Methods). PSR = plant species richness; Pi = inorganic P, PUE = phosphorus use efficiency (inverse of plant P concentrations), C_{org} = organic carbon concentrations in soil, P_{mic} = microbial P, F = fungi, B = bacteria, PAse = phosphatase activity in soil.

**Biodiversity Experiment**	(log) PSR	(log) Bioavai-lable Pi	(log) Plant biomass	(log) Plant P conc	PUE	N:P ratio	Plant P stocks	Plant P exploi-tation	Grass contri-bution	Herb contri-bution	Legume contri-bution	pH	(log) C_{org} conc	C_{org} stocks	Mode-rately labile Pi	Mineral P	(log) Microbial P conc	Microbial P stocks	Microbial P ex-ploitation	Total P exploi-tation	Cmic: Pmic	Fungi	F:B ratio	AMF SR	(log) Relative AMF ab. abundance
(log) Bioavailable Pi	0.22																								
(log) Plant biomass	0.52***	0.27*																							
(log) Plant P conc	0.02	0.31**	-0.06																						
PUE	-0.04	-0.26*	0.06	-0.98***																					
N:P ratio	-0.16	-0.41***	-0.34**	-0.33**	0.29*																				
Plant P stocks	0.55***	0.24*	0.73***	0.08	-0.09	-0.27*																			
Plant P exploitation	0.35**	-0.17	0.64***	-0.08	0.06	-0.13	0.80***																		
Grass contribution	-0.04	0.21	0.17	-0.61***	0.64***	-0.08	-0.05	-0.01																	
Herb contribution	0.13	0.05	0.09	0.57***	-0.56***	-0.42***	0.16	0.06	-0.66***																
Legume contribution	-0.12	-0.30**	-0.31**	0.00	-0.05	0.61***	-0.14	-0.07	-0.34**	-0.48***															
pH	-0.04	-0.34**	-0.23*	-0.12	0.10	0.25*	0.00	0.11	-0.06	-0.10	0.20														
(log) C_{org} conc	0.40***	0.64***	0.30**	0.18	-0.14	-0.23*	0.39***	0.07	0.08	0.04	-0.14	-0.29*													
C_{org} stocks	0.29*	0.68***	0.30*	0.24*	-0.19	-0.28*	0.35**	0.01	0.10	0.03	-0.15	-0.34**	0.96***												
Moderately labile Pi	0.20	0.97***	0.24*	0.30**	-0.26*	-0.38***	0.24*	-0.17	0.18	0.07	-0.31**	-0.38***	0.68***	0.71***											
Mineral P	-0.02	0.57***	0.07	0.14	-0.09	-0.02	0.10	-0.17	0.10	-0.01	-0.10	0.03	0.42***	0.47***	0.58***										
(log) Microbial P conc	0.38***	0.73***	0.28*	0.32**	-0.28*	-0.38***	0.28*	-0.12	0.11	0.06	-0.20	-0.28*	0.68***	0.69***	0.71***	0.38***									
Microbial P stocks	0.26*	0.59***	0.20	0.32**	-0.29*	-0.35**	0.18	-0.16	0.06	0.09	-0.17	-0.27*	0.51***	0.54***	0.58***	0.30**	0.94***								
Microbial P exploitation	0.10	-0.22	-0.12	0.05	-0.05	-0.03	-0.08	-0.10	-0.12	0.05	0.07	0.22	-0.06	-0.07	-0.25*	-0.10	0.42***	0.56***							
Total P exploitation	0.19	-0.24*	0.12	0.04	-0.05	-0.07	0.23	0.24*	-0.16	0.11	0.05	0.26*	-0.04	-0.06	-0.29*	-0.12	0.37**	0.49***	0.94***						
Cmic:Pmic	-0.08	-0.38**	-0.05	-0.13	0.08	0.12	0.16	0.33**	-0.26*	0.24	0.00	0.33**	-0.28*	-0.31*	-0.36**	-0.15	-0.46***	-0.39**	-0.05	0.11					
Fungi	0.18	0.18	0.12	0.10	-0.11	-0.17	0.07	0.02	-0.08	0.24*	-0.21	-0.40***	0.24*	0.24*	0.20	-0.03	0.27*	0.30**	0.03	0.00	-0.08				
F:B ratio	0.01	-0.11	0.07	0.11	-0.13	-0.17	-0.10	-0.02	-0.06	0.27*	-0.27*	-0.32**	-0.31**	-0.25*	-0.12	-0.23*	-0.10	-0.02	0.01	-0.01	-0.08	0.60***			
AMF SR	0.22	0.10	0.11	0.10	-0.09	-0.08	0.06	-0.08	-0.03	0.00	0.04	0.01	0.17	0.18	0.08	0.00	0.18	0.15	0.07	0.08	-0.40**	0.11	0.12		
(log) Relative AMF abundance	0.11	0.06	0.04	0.07	-0.05	-0.04	0.04	-0.11	-0.09	0.04	0.05	0.04	0.09	0.09	0.04	0.03	0.13	0.13	0.11	0.11	-0.29*	0.14	0.13	0.90***	
PAse	0.59***	0.35**	0.40***	0.07	-0.05	-0.15	0.44***	0.23	0.02	0.07	-0.11	-0.23*	0.72***	0.65***	0.39***	0.13	0.56***	0.41***	0.06	0.11	-0.16	0.25*	-0.17	0.17	0.07

Supplementary Table 2: Correlation (Pearson) matrix of variables in agricultural grasslands. Significant negative and positive relationships in blue and orange, respectively (hue refers to the closeness of the relationship). Asterisks refer to the level of significance (*** $p < 0.001$, ** $p < 0.01$, * $p < 0.05$). The Benjamini & Hochberg correction was applied for the multiple testing (see Methods). Pi = inorganic P, PUE = phosphorus use efficiency (inverse of plant P concentrations), C_{org} = organic carbon concentrations in soil, P_{mic} = microbial P, F = fungi, B = bacteria, PAse = phosphatase activity in soil, LUI = land use intensity (see Methods).

**Agricultural grasslands**	LUI	(log) PSR	(log) Bioavai-lable Pi	(log) Plant biomass	Plant P conc	PUE	N:P ratios	Plant P stocks	Plant P exploi-tation	Grass contri-bution	Herb contri-bution	Legume contri-bution	pH	(log) Corg conc	Corg stocks	Mode-rately labile Pi	Mineral P	(log) Microbia l P conc	Micro-bial P stocks	Micro-bial P exploitat ion	Total P exploi-tation	Nmic:	Agricult ural grasslan ds	LUI	(log) PSR	(log) Bioavai-lable Pi
(log) PSR	-0.66***																									
(log) Bioavailable Pi	0.52***	-0.60***																								
(log) Plant biomass	0.63***	-0.62***	0.62***																							
Plant P conc	0.62***	-0.61***	0.75***	0.61***																						
PUE	-0.56***	0.52***	-0.66***	-0.56***	-0.92***																					
N:P ratios	-0.45***	0.47***	-0.62***	-0.59***	-0.61***	0.53***																				
Plant P stocks	0.60***	-0.59***	0.56***	0.88***	0.57***	-0.47***	-0.53***																			
Plant P exploitation	0.54***	-0.47***	0.38***	0.79***	0.41***	-0.38***	-0.43***	***																		
Grass contribution	0.30**	-0.34***	0.24*	0.34***	0.22*	-0.20*	-0.22*	0.33***	0.44***																	
Herb contribution	-0.29**	0.32**	-0.26**	-0.34***	-0.23*	0.22*	0.25*	-0.29**	-0.35***	-0.90***																
Legume contribution	-0.05	0.05	0.04	-0.02	0.05	-0.05	-0.06	-0.10	-0.27**	-0.42***	-0.02															
pH	-0.04	0.21*	0.01	-0.14	-0.12	0.01	0.21*	-0.19	0.04	-0.11	0.15	-0.08														
(log) Corg conc	0.05	-0.11	0.09	0.00	0.16	-0.02	0.08	0.13	-0.17	-0.04	0.04	0.00	-0.29**													
Corg stocks	0.24*	-0.20	0.20	0.04	0.30**	-0.15	-0.01	0.17	-0.17	-0.10	0.06	0.12	-0.35***	0.93***												
Moderately labile Pi	0.33**	-0.48***	0.64***	0.45***	0.65***	-0.47***	-0.40***	0.49***	0.07	0.08	-0.11	0.09	-0.47***	0.50***	0.57***											
Mineral P	0.37***	-0.21*	0.51***	0.36***	0.39***	-0.36***	-0.35***	0.35***	0.33**	0.16	-0.12	-0.10	0.31**	-0.01	0.03	0.25*										
(log) Microbial P conc	0.08	-0.03	0.01	0.01	0.10	0.01	0.05	-0.04	-0.19	-0.08	0.08	0.02	-0.26**	0.65***	0.61***	0.27**	-0.02									
Microbial P stocks	0.24*	-0.11	0.11	0.06	0.18	-0.09	-0.07	-0.03	-0.15	-0.10	0.07	0.13	-0.26*	0.37***	0.46***	0.23*	0.03	0.89***								
Microbial P exploitation	-0.35***	0.46***	-0.55***	-0.45***	-0.47***	0.41***	0.53***	-0.44***	-0.16	-0.18	0.23*	-0.12	0.35***	-0.07	-0.16	-0.62***	-0.19	0.29**	0.28**							
Total P exploitation	-0.20	0.34**	-0.45***	-0.24*	-0.36***	0.31**	0.41***	-0.21*	0.12	-0.06	0.14	-0.20	0.37***	-0.11	-0.21	-0.60***	-0.10	0.24*	0.24*	0.96***						
Nmic:Pmic	0.18	-0.19	0.36***	0.25*	0.19	-0.21*	-0.14	0.34***	0.30**	0.10	-0.10	-0.02	0.35***	0.05	0.06	0.14	0.37***	-0.52***	-0.57***	-0.44***	-0.36***					
Fungi	-0.26**	0.28**	-0.35***	-0.30**	-0.31**	0.30**	0.41***	-0.24*	-0.27**	-0.29**	0.28**	0.05	-0.03	0.45***	0.34**	-0.12	-0.10	0.32**	0.14	0.32**	0.25*	-0.10				
(log) AMF relative abundance	-0.33***	0.26**	-0.41***	-0.30**	-0.30**	0.26**	0.27**	-0.23*	-0.12	-0.06	0.11	-0.15	0.08	0.05	0.03	-0.24*	-0.36***	0.01	-0.07	0.36***	0.32**	-0.19	0.06			
AMF SR	-0.06	0.17	-0.14	-0.09	-0.13	0.00	0.07	-0.15	0.09	0.06	-0.01	-0.14	0.33***	-0.38***	-0.35***	-0.43***	-0.07	-0.25*	-0.15	0.35***	0.37***	-0.05	-0.19	0.35***		
F:B ratio	-0.43***	0.50***	-0.52***	-0.45***	-0.50***	0.38***	0.46***	-0.47***	-0.24*	-0.26**	0.28**	-0.03	0.33***	-0.41***	-0.48***	-0.57***	-0.15	-0.34***	-0.31**	0.42***	0.35***	-0.13	0.49***	0.15	0.25*	
PAse	0.17	-0.33***	0.16	0.21*	0.29**	-0.13	-0.17	0.25*	0.01	0.21*	-0.24*	0.03	-0.74***	0.47***	0.49***	0.54***	-0.27**	0.37***	0.31**	-0.30**	-0.30**	-0.27**	-0.06	0.05	-0.32**	-0.52***

Supplementary Table 3: Primers used for the identification of fungi in the biodiversity experiment and the agricultural grasslands

Primer	Sequence	Reference
Biodiversity experiment		
FR 1	5’-AICCATTCAATCGGTAIT -3	Kuramae et al., 2013[1]
modified version of FF390	5′-CGWTAACGAACGAGACCT-3′	Kuramae et al., 2013[1]
Agricultural grasslands		
GlomerWT0	GDWTCATTCAAATTTCTGCCCTAT	Wubet et al., 2006[2]
Glomer1536	RTTGCAATGCTCTATCCCCA	Wubet et al., 2006[2]
NS31	TTGGAGGGCAAGTCTGGTGCC	Simon et al., 1992[3]
AML2	GAACCCAAACACTTTGGTTTCC	Lee et al., 2008[4]

Supplementary Table 4: Goodness-of-fit statistics for conceptual and final structural equation models testing the P exploitation in the biodiversity experiment and the agricultural grasslands. Specifically, the table gives the Fisher's C test statistic, the Chi-squared test degrees of freedom (df), the significance derived from a Chi-squared distribution (P-value).

	Model	Fisher's C	*df*	*P*-value
Biodiversity experiment (N = 70)	initial	190.4	20	0.000
	final	7.1	14	0.930
Agricultural grasslands (N = 91)	initial	76.9	24	0.000
	final	12.2	12	0.432

Supplementary Table 5: The explained variance for each endogenous variable fitted in the final structural equation models (Table S1) for the biodiversity experiment and the agricultural grasslands. Marginal (i.e. explained by fixed effects alone) and conditional (i.e. explained by fixed effects and random effects) pseudo-R-squared values are given[5].

	Biodiversity experiment		Agricultural grasslands	
Response	R^2_m	R^2_c	R^2_m	R^2_c
Plant species richness			0.41	0.41
C_{org} stocks	0.08	0.57	0.04	0.65
AMF species richness	0.05	0.16	0.01	0.35
Relative AMF abundance	0.02	0.02	0.11	0.11
Plant P stocks	0.37	0.40	0.45	0.48
Microbial P stocks	0.41	0.42	0.26	0.27
Total P exploitation	0.34	0.43	0.32	0.43

Supplementary Table 6: Standardised estimates (scaled) and their respective *P* values of all relations included in the final structural equation models testing the P exploitation in the biodiversity experiment and in the agricultural grasslands (Fig. 1 in the main manuscript). Numbers in bold indicate a significant relationship between response and predictor variable. Correlated errors between variables are indicated by ~~. They indicate that the relationship between the two variables is not presumed to be causal and unidirectional.

Response	Predictor	Std.estimate	*P* value
**Biodiversity experiment**			
**(log) C_{org} stocks**	**(log) Plant species richness**	**0.293**	**0.001**
**AMF species richness**	**(log) Plant species richness**	**0.232**	**0.043**
(log) Relative AMF abundance	(log) Plant species richness	0.133	0.272
(log) Plant P stocks	AMF species richness	-0.253	0.325
(log) Plant P stocks	(log) Relative AMF abundance	0.200	0.414
(log) Plant P stocks	(log) C_{org} stocks	0.180	0.120
**(log) Plant P stocks**	**(log) Plant species richness**	**0.564**	**< 0.001**
(log) Microbial P stocks	AMF species richness	0.023	0.920
(log) Microbial P stocks	(log) Relative AMF abundance	0.073	0.746
**(log) Microbial P stocks**	**(log) C_{org} stocks**	**0.636**	**< 0.001**
**(log) Total P exploitation**	**(log) Microbial P stocks**	**0.703**	**< 0.001**
**(log) Total P exploitation**	**(log) Plant P stocks**	**0.260**	**0.014**
**(log) Total P exploitation**	**(log) C_{org} stocks**	**-0.564**	**< 0.001**
**~~(log) Relative AMF abundance**	**~~AMF species richness**	**0.934**	**< 0.001**
**Agricultural grasslands**			
**(log) plant species richness**	**Land use intensity index**	**-0.640**	**< 0.001**
(log) C_{org} stocks	(log) plant species richness	0.019	0.848
**(log) C_{org} stocks**	**Land use intensity index**	**0.241**	**0.017**
AMF species richness	(log) plant species richness	0.107	0.259
(log) AMF relative abundance	(log) plant species richness	0.061	0.642
**(log) AMF relative abundance**	**Land use intensity index**	**-0.291**	**0.029**
Plant P stocks	AMF species richness	-0.061	0.533
Plant P stocks	(log) AMF relative abundance	0.020	0.828
Plant P stocks	(log) C_{org} stocks	0.018	0.873
**Plant P stocks**	**Land use intensity index**	**0.451**	**< 0.001**
**Plant P stocks**	**(log) Microbial P stocks**	**-0.205**	**0.028**
**Plant P stocks**	**(log) plant species richness**	**-0.308**	**0.004**
(log) Microbial P stocks	AMF species richness	0.057	0.603
(log) Microbial P stocks	(log) AMF relative abundance	-0.061	0.547
**(log) Microbial P stocks**	**(log) C_{org} stocks**	**0.544**	**< 0.001**

**(log) Total P exploitation**	**(log) Microbial P stocks**	**0.524**	**< 0.001**
(log) Total P exploitation	Plant P stocks	0.107	0.362
(log) Total P exploitation	Land use intensity index	-0.035	0.785
**(log) Total P exploitation**	**(log) AMF relative abundance**	**0.308**	**0.001**
**(log) Total P exploitation**	**(log) C_{org} stocks**	**-0.283**	**0.027**
**(log) Total P exploitation**	**(log) plant species richness**	**0.248**	**0.035**
**~~(log) AMF relative abundance**	**~~AMF species richness**	**0.452**	**< 0.001**

12

Supplementary Figure 1: Hypothesis model biodiversity experiment. Blue arrows display relationships with positive slopes. Expectations according to the hypotheses can be found in Table S1. The SEM did not match with the data: Fisher's C = 190.44, p = 0, df = 20. P = phosphorus, C_{org} = organic carbon. Photo credit: The Jena Experiment.

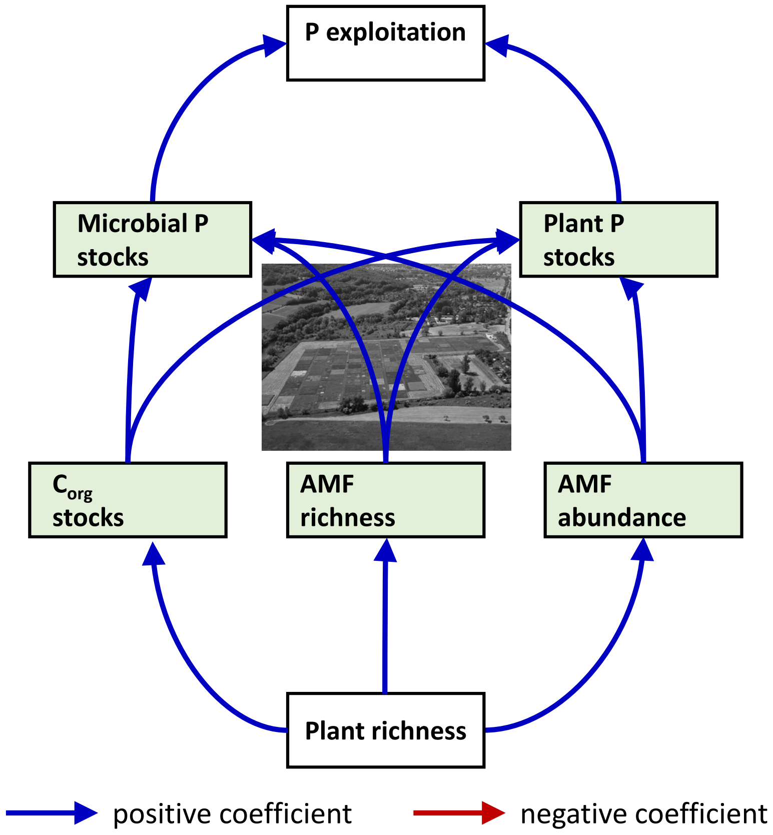

Supplementary Figure 2: Hypothesis model agricultural grasslands according to the final model for the biodiversity experiment. Blue and red arrows display relationships with positive and negative slopes, respectively. Expectations according to the hypotheses can be found in Table S1. Round-shaped paths refer to the (driving) role of biodiversity while square paths indicate the role of management. The SEM did not match with the data: Fisher's C = 76.88, p = 0, df = 24. P = phosphorus, C_{org} = organic carbon. Photo credit: Jörg Hailer.

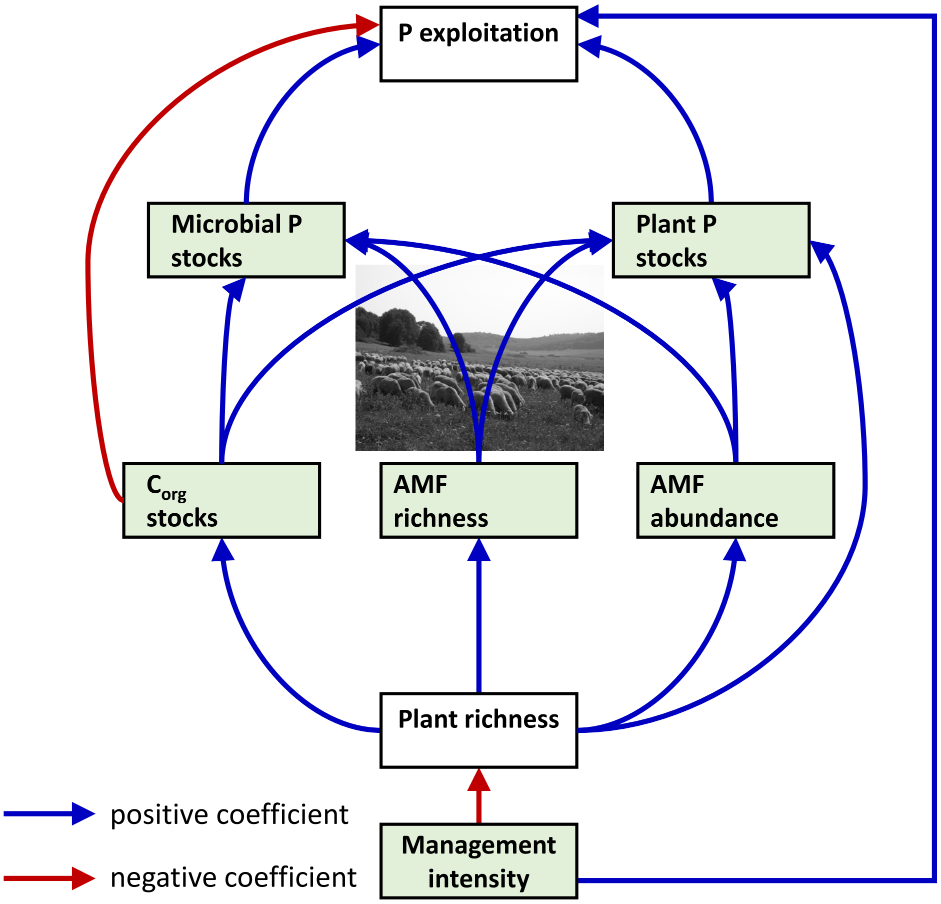

Supplementary Figure 3: Regression of the number of plant species in a 9-m² plot on the number of plant species in a 16-m² plot assessed in a survey of nested subplots of selected plots in agricultural grasslands (*n* = 18). The slope of the regression was used as a factor to downscale plant species richness in agricultural grasslands according to the area used for the assessment of plant species richness in the biodiversity experiment.

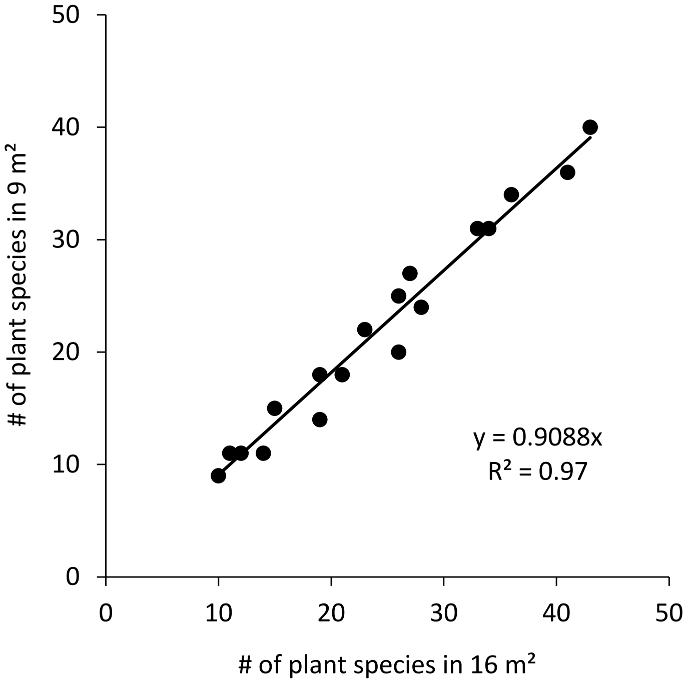

Supplementary Reference

1. Kuramae EE*, et al.* Tracking Fungal Community Responses to Maize Plants by DNA- and RNA-Based Pyrosequencing. *Plos One* **8**, 8 (2013).

2. Wubet T, Weiss M, Kottke I, Oberwinkler F. Two threatened coexisting indigenous conifer species in the dry Afromontane forests of Ethiopia are associated with distinct arbuscular mycorrhizal fungal communities. *Canadian Journal of Botany-Revue Canadienne De Botanique* **84**, 1617-1627 (2006).

3. Simon L, Lalonde M, Bruns TD. Specific amplification of 18S fungal ribosomal genes from vesicular-arbuscular endomycorrhizal fungi colonizing roots. *Appl Environ Microbiol* **58**, 291-295 (1992).

4. Lee J, Lee S, Young JPW. Improved PCR primers for the detection and identification of arbuscular mycorrhizal fungi. *FEMS Microbiol Ecol* **65**, 339-349 (2008).

5. Lefcheck JS. PIECEWISESEM: Piecewise structural equation modelling in R for ecology, evolution, and systematics. *Methods Ecol Evol* **7**, 573-579 (2016).
