# Supplementary material for: Above- and belowground biodiversity jointly tighten the P cycle in agricultural grasslands
Source: Nat Commun. 2021 Jul 21;12:4431. doi: 10.1038/s41467-021-24714-4 (PMC8295381; doi:10.1038/s41467-021-24714-4)
Supplement: Supplementary file 3 — Reporting Summary [file 41467_2021_24714_MOESM3_ESM.pdf]

nature research

Corresponding author(s): Yvonne Oelmann

Last updated by author(s): Jun 7, 2021

Reporting Summary

Nature Research wishes to improve the reproducibility of the work that we publish. This form provides structure for consistency and transparency in reporting. For further information on Nature Research policies, see our Editorial Policies and the Editorial Policy Checklist.

Statistics

For all statistical analyses, confirm that the following items are present in the figure legend, table legend, main text, or Methods section.

n/a	Confirmed	
☐	☒	The exact sample size (n) for each experimental group/condition, given as a discrete number and unit of measurement
☐	☒	A statement on whether measurements were taken from distinct samples or whether the same sample was measured repeatedly
☐	☒	The statistical test(s) used AND whether they are one- or two-sided<br>*Only common tests should be described solely by name; describe more complex techniques in the Methods section.*
☐	☒	A description of all covariates tested
☐	☒	A description of any assumptions or corrections, such as tests of normality and adjustment for multiple comparisons
☐	☒	A full description of the statistical parameters including central tendency (e.g. means) or other basic estimates (e.g. regression coefficient) AND variation (e.g. standard deviation) or associated estimates of uncertainty (e.g. confidence intervals)
☐	☒	For null hypothesis testing, the test statistic (e.g. F, t, r) with confidence intervals, effect sizes, degrees of freedom and P value noted<br>*Give P values as exact values whenever suitable.*
☒	☐	For Bayesian analysis, information on the choice of priors and Markov chain Monte Carlo settings
☐	☒	For hierarchical and complex designs, identification of the appropriate level for tests and full reporting of outcomes
☐	☒	Estimates of effect sizes (e.g. Cohen's d, Pearson's r), indicating how they were calculated

Our web collection on statistics for biologists contains articles on many of the points above.

Software and code

Policy information about availability of computer code

Data collection: no software was used

Data analysis: The AMF sequences were processed using a customized bioinformatic pipeline following MOTHUR SOP (using Version 1.39.5) as implemented in DeltaMP (https://github.com/lentendu/DeltaMP).
Pearson correlations were calculated applying the corr.test function in the R library psych (R version 4.0.5). The Pearson correlations are corrected for multiple inference using the Benjamini & Hochberg correction. Using the R library piecewiseSEM, confirmatory path analyses were applied to test the causal relationships between plant diversity and P exploitation in the biodiversity experiment and in the agricultural grasslands and how land-use intensity impacts this relation in the latter. In order to test whether the conceptual models adequately represent the measured data Fisher's C test statistic was used. To obtain adequate models the conceptual models were updated stepwise by including missing paths as indicated by the tests of directed separation. We did not use custom algorithm that would require code deposition.

For manuscripts utilizing custom algorithms or software that are central to the research but not yet described in published literature, software must be made available to editors and reviewers. We strongly encourage code deposition in a community repository (e.g. GitHub). See the Nature Research guidelines for submitting code & software for further information.

Data

Policy information about availability of data

All manuscripts must include a data availability statement. This statement should provide the following information, where applicable:

- Accession codes, unique identifiers, or web links for publicly available datasets
- A list of figures that have associated raw data
- A description of any restrictions on data availability

This work is based on data from several projects of the Biodiversity Exploratories programme (DFG Priority Program 1374) and The Jena Experiment (DFG FOR 456,

500). The data used for analyses are publicly available from the Biodiversity Exploratories Information System (https://doi.org/10.17616/R32P9Q) and the jexis database (https://jexis.idiv.de/), respectively, or will become publicly available after an embargo period of five years from the end of data assembly to give the owners and collectors of the data time to perform their analysis. The raw AMF Illumina sequences for the Biodiversity Exploratories have been deposited in the National Center for Biotechnology Information (NCBI) Sequence Read Archive (SRA) under BioProject accession number PRJNA706003. Microbial community data of the Jena Experiment have been archived in the Pangaea database (https://doi.org/10.1594/pangaea.874990). Any other relevant data are available from the corresponding author upon reasonable request.

Field-specific reporting

Please select the one below that is the best fit for your research. If you are not sure, read the appropriate sections before making your selection.

☐ Life sciences ☐ Behavioural & social sciences ☒ Ecological, evolutionary & environmental sciences

For a reference copy of the document with all sections, see nature.com/documents/nr-reporting-summary-flat.pdf

Ecological, evolutionary & environmental sciences study design

All studies must disclose on these points even when the disclosure is negative.

Study description

The study includes two large research consortia: (1) a grassland biodiversity experiment and (2) agricultural grasslands. (1) Grassland biodiversity experiment: Our data originate from The Jena Experiment (http://www.the-jena-experiment.de). The main experiment comprises 78 plots. Each plot contains a specific combination of plant species (1, 2, 4, 8, 16 species) that belong to different numbers (1, 2, 3, 4) of plant functional groups (grasses, [non-leguminous] small herbs, [non-leguminous] tall herbs, legumes). The species were chosen from a pool of 60 species typically found in mesic Molinio-Arrhenatheretea meadows. Each plant species richness level had 16 replicates except for 14 mixtures with 16 species. (2) Agricultural grasslands: We studied long-established, agriculturally managed (i.e. not experimentally assembled) grasslands in an interdisciplinary, large-scale and long-term programme, the Biodiversity Exploratories (http://www.biodiversity-exploratories.de). The design of the Biodiversity Exploratories is described in detail by Fischer et al. 2010. We used 100 grassland plots (50 in each of Schwäbische Alb and Hainich-Dün) which can be classified as meadows (mown one to four times per year but not grazed), pastures (grazed but not mown), and mown pastures (both mown and grazed). Plots were selected to represent a gradient of management intensity with different fertilisation, frequency of mowing, and livestock units.

Research sample

The sample unit is the soil as well as the community of a grassland plot, the latter considering the plant and arbuscular mycorrhizal fungi (AMF) community. It is characterized by measures of organic carbon concentrations, phosphorus concentrations (soil, plants) and biomass, species, amplicon sequence variants or operational taxonomic units numbers, and relative abundance (plants, AMF). A sample unit is considered to represent the population of the different groups at our study sites which measured 50 m x 50 m.

Sampling strategy

Plant species richness and biomass: The plots of the biodiversity experiment were split in different subplots with a core area of approximately 43.5 m^2. Realised target species richness was determined on a 9 m2 (3 m x 3 m) area.

In agricultural grasslands, vascular plant species richness was assessed on a 16 m2 (4 m x 4 m) area by sampling all species . In order to account for the difference in survey area between experimental and agricultural grasslands, a separate survey of 9 m^2 subplots nested within the 16 m^2 subplots was conducted in April and May 2018 in a selected number of plots in the agricultural grasslands ($n = 18$). Species richness differed significantly between 9 m2 and 16 m2 subplots (mean number of species ± standard error; 9 m2: 22 ± 2; 16 m2: 24 ± 2; paired t-test: $T = -5.15$; $p < 0.001$). Accordingly, we used a scaling factor of 0.91 i.e., the slope of the regression of species richness on 9 m^2 on species richness on 16 m2 (Fig. S5), to adjust species richness of all agricultural grasslands to the area of species richness measurements in the biodiversity experiment.

In the biodiversity experiment, aboveground plant biomass was collected on all plots within a frame (0.2 x 0.5 m, height 0.03 m) at two random locations per plot and sorted by target species, weeds and detached dead plant material. Biomass was extrapolated to 1 m2. In agricultural grasslands, biomass was sampled on 2 m2 as mixed samples of eight randomly placed quadrats of 0.25 m2 in close proximity to the vegetation records. Temporary fences ensured that biomass was sampled at peak standing crop but without any effect of possible mowing or grazing events. Detached dead material was excluded from biomass sampling. Biomass was assessed on a dry-weight basis (drying at 70-80 °C for 48 h) in the experimental and agricultural grasslands.

Soil sampling: Soil in the biodiversity experiment was sampled for analyses of Corg concentrations and bulk density. In each plot, three soil cores were taken to a depth of 0.3 m using a split-tube sampler (4.8 cm diameter). Soil cores were segmented into 5 cm-depth sections and pooled per depth sections and plot. We used the mean Corg concentrations of the upper three intervals (0 to 0.15 m). For the measurement of P in soil of the biodiversity experiment, nine soil cores per plot with a diameter of 0.02 m were taken at a depth of 0 to 0.15 m and combined to a composite sample considered representative for the plot.

In agricultural grasslands, soil samples were taken along two orthogonal transects of 20 m. Sampling points were shifted by 0.5 m in 2014 compared with 2011 to avoid an overlap of sampling positions. In each plot, 14 samples from 0 to 0.1 m soil depth were taken using core augers (diameter ~ 52 mm). Samples were mixed, cooled and transported to a field lab where they were sieved (< 2 mm), all within eight hours of sampling. All measurements described below for agricultural grasslands were done on aliquots of samples of these joint sampling campaigns.

No statistical methods were used to predetermine sample size.

Data collection

Air-dry plant material sampled in the experimental and agricultural grasslands was ground with a mill using a 0.5-mm screen for chemical analyses. Nitrogen concentration of plant material was measured in ground samples with an elemental analyser for the biodiversity experiment and near-infrared spectroscopy (NIRS) for the agricultural grasslands. Plant samples were digested in a microwave with concentrated nitric acid and hydrogen peroxide54 and P concentrations were determined by inductively-coupled plasma optical emission spectrometry (ICP-OES). In 2011, P concentrations were measured by NIRS in biomass of the agricultural grasslands. We multiplied the latter data by 0.9844 to match with the digestion method based on data of the year 2009 for which both the digestion and NIRS method had been applied ($P_{digestion} = 0.9844 \times P_{NIRS}$, $r = 0.80$, $p < 0.001$, $n = 98$). Plant samples collected in 2014 in the agricultural grasslands were analysed for P concentrations by means of an X-ray fluorescence spectrometer. We can exclude a methodological shift in plant P concentrations between years, because digestion followed by ICP-OES analyses and

XRF analyses were shown to match well. Nitrogen:P ratios in plant material were calculated on an elemental mass basis. We calculated P stocks in aboveground biomass by multiplying biomass [g m-2] harvested in May with their P concentrations [mg g-1] and with the number of mowing events. In this way, we likely overestimated the absolute values of aboveground plant P stocks. But at the same time, we ensured comparability between experimental and agricultural grasslands because, for the latter, plant material of mowing events later than May was not available.

In the experimental and agricultural grasslands, soil pH values were determined with a glass electrode in a 1:2.5 soil:0.01 M $CaCl_2$ water suspension. Total C concentrations were determined on ground air-dry samples by an elemental analyser. Organic C concentrations were calculated by subtracting inorganic C concentrations (determined after removal of Corg at 450 °C in a muffle furnace) from total C concentration. Soil bulk density was calculated by weighing a 100-cm^3 core after drying the soil at 40°C.

In the experimental and agricultural grasslands, various P fractions in soil were measured. The sequential extraction scheme had four steps (bioavailable P ($NaHCO_3$ extractable), moderately labile P (NaOH extractable), mineral P (HCl extractable) and occluded P (H_2SO_4 extractable)). The bioavailable P fraction comprises $H_2PO_4^-$/HPO_4^{2-} ions in soil solution and those weakly adsorbed to mineral surfaces. More strongly adsorbed P to iron and aluminium oxides and (oxy)hydroxides is recovered in the moderately labile fraction. The mineral P fraction contains P bound in apatite and other Ca phosphates. Occluded P refers to a fraction that is spatially or chemically protected against P transformation reactions. In all extraction solutions, Pi concentrations were analysed using the ammonium molybdate-ascorbic acid blue method and measured with a continuous flow analyser. Total dissolved P concentrations in $NaHCO_3$- and NaOH-extracts were measured with an ICP-OES. For the labile and moderately labile fractions ($NaHCO_3$-P, NaOH-P), organic P concentrations were calculated by subtracting Pi from total dissolved P concentrations in the extracts.

In the experimental and agricultural grasslands, microbial P was measured . Three subsamples of each soil sample were prepared by adding deionised water and one anion-exchange membrane. One of the three subsamples was additionally mixed with hexanol as fumigation reagent (PHex) and one with a P spike (PSpike), while nothing was added to the last subsample (PH2O). Nitric acid was used to exchange the P adsorbed onto the membranes. Phosphate concentrations in solutions were measured with a continuous flow analyser. We calculated microbial P concentrations as the difference between hexanol-fumigated and non-fumigated samples and accounted for P retention during extraction by including the P spike. Because the calculated microbial P concentrations underestimate the amount of P stored in microbial biomass because of fumigation/extraction efficiency constraints e.g., with respect to gram-positive bacteria, we divided calculated microbial P concentrations by 0.4. We calculated stocks of P fractions and microbial P in soil based on bulk density [g m-2 (0.15 m soil depth)-1] and multiplication with the respective P concentrations [mg g-1]. Phosphorus exploitation was calculated as the contribution [%] of organismic P stocks (either in the aboveground part of plants or microbes) to the sum of bioavailable P stocks (both organismic P stocks + labile P stocks + moderately labile P stocks). The aboveground plant P stocks represents the plant demand of P that is removed with the harvest(s) each year. It is reasonable to assume that microbial P stocks in soil can also be regarded as the annual microbial P demand since it has been shown that the microbial P stock in soil turns over once every growing season irrespective of management.

In the biodiversity experiment, phosphomonoesterase (PAse) activity was measured. For each soil sample, one replicate and one blank value were included in the laboratory study. We incubated soil samples with p-nitrophenylphosphate as an organic substrate for enzyme activity (pH = 11). p-nitrophenylphosphate was added to blanks only after incubation. Directly after filtration, p-nitrophenol concentrations were measured with a spectrophotometer. In agricultural grasslands, PAse activity was determined by fluorescence measures in a buffered solution of pH 6.1.

In the biodiversity experiment, the fungal-to-bacterial ratio was assessed by applying the phospholipid fatty acids (PLFA) method. Within 48 h after sampling, the soil was kept at 4 °C, sieved to 2 mm, remains of roots were manually removed and the samples were stored at −20 °C until further sample processing. PLFA were extracted. As an indicator for fungal PLFA 18:2ω6,9 was used. The bacterial PLFA was calculated as sum of the PLFA markers 14:0i, 15:0i, 15:0a, 16:0i, c16:1ω7c, 17:1, 17:0i, 17:0a and 18:1ω7. Furthermore, AMF species richness and relative abundance in the biodiversity experiment was investigated in soil samples (0-15 cm depth) collected in 2010 and analysed using DNA extraction and amplicon sequencing by 454-pyrosenquencing. We amplified 18S rRNA gene fragments from fungi and protists with primer FR 1 and the modified version of FF390 designed to also include the Glomeromycota comprising AMF. In agricultural grasslands, AMF were identified based on DNA extraction. DNA was extracted from soil of each plot using the MO BIO Power Soil DNA isolation kit (MO BIO Laboratories, Carlsbad, CA, USA) following the manufacturer's protocol. Afterwards we used a nested PCR approach to amplify fungal 18S-rDNA by using the primer pairs GlomerWT0/Glomer1536 and NS31/AML2, containing the Illumina adapter sequences. PCR products were then purified, cleaned and sequenced using Illumina MiSeq. The AMF sequences were processed using a customized bioinformatic pipeline following MOTHUR SOP (using Version 1.39.5) as implemented in DeltaMP (https://github.com/lentendu/DeltaMP) . The taxonomical assignment was done against the MaarjAM database (https://maarjam.botany.ut.ee/). AMF OTUs were merged according to VT assignment. OTU assigned only to genus level were kept as OTUs. Only those AMF appearing on more than five plots were considered. AMF species richness was calculated as the number of species including OTUs. The relative abundance of AMF was calculated by relating the reads per species/OTU to the total sum of reads across all plots. AMF appearing on less than five plots had low relative abundances (< 1%) and thus, were considered to play a negligible role. Accordingly, only those AMF appearing on more than five plots were included. AMF species richness was calculated as the number of species including OTUs. The relative abundance of AMF was calculated by relating the reads per species/OTU to the total sum of reads across all plots.

Timing and spatial scale

Biodiversity experiment:
- Realised target species richness and biomass in May 2014
- Soil in the biodiversity experiment in April 2014. For the measurement of P in soil of the biodiversity experiment, sampling took place before the growing season started i.e., in September 2013. Although these samples were not taken during the identical period of time as for the agricultural grasslands (see below), we tested whether the results are applicable to the growing season of 2014. First, bioavailable Pi concentrations in soil were closely correlated between years (September 2013 and October 2014; $r = 0.88$, $p < 0.001$, $n = 79$). Second, resin-extractable P concentrations on samples of the 2013 campaign measured in the laboratory matched with P released under field conditions in May 2014 ($r = 0.40$, $p < 0.001$, $n = 78$).

Agricultural grasslands:
- Vascular plant species richness and aboveground biomass from mid May to mid June in 2011 and 2014.
- Soil samples in May 2011 and May 2014.

Spatial scale: All these data were collected within a 43.5 m^2 area for 76 plots (biodiversity experiment) and in a 50 m x 50 m area, in 100 grassland plots (agricultural grasslands). These agricultural grassland plots were chosen to cover a wide gradient of land-use intensity.

Data exclusions	No data were excluded from analyses.
Reproducibility	The biodiversity experiment is a large field-scale study and thus it is impossible to reproduce the experiment. Nevertheless, there are several grassland biodiversity experiments worldwide (e.g. in Cedar Creek, USA) which closely cooperate and can be compared. The agricultural grasslands include three regions where the identical experimental design is realized (and thus, reproduced).
Randomization	Biodiversity Experiment: Species and species compositions were allocated randomly across the field site (while the systematic variation in soil texture was accounted for by a block design). Agricultural grasslands: the plots were allocated randomly in the field within the given management intensity classes.
Blinding	Since the study did not use living organisms, blinding does not apply.

Did the study involve field work? [x] Yes [ ] No

Field work, collection and transport

Field conditions	Biodiversity experiment: Mean annual air temperature is 9.9°C, and mean annual precipitation amounts to 610 mm. The soil is an Eutric Fluvisol developed from up to 2 m-thick fluvial sediments that are almost free of stones. Sediments largely originate from the same geological series (Pleistocene loess on limestone [Anisian/Ladinian] of the Middle Triassic) as described for one of the agricultural grassland regions (Hainich-Dün, see below). The systematic variation in soil texture as a consequence of fluvial dynamics is considered in the experimental design by arranging the experimental plots in four blocks at different distance to the river. The study site was converted from grassland to arable land in the early 1960s and used for cropping until the establishment of the experiment in 2002.<br>Agricultural grasslands:The Biodiversity Exploratories comprise three study regions in Germany (Schwäbische Alb, Hainich-Dün, and Schorfheide-Chorin). We aimed to disentangle biodiversity and management effects and therefore, had to remove additional confounding factors such as geologic parent material (calcareous versus non-calcareous). Because of substantial differences in the geologic parent material and of P fractions in soil, we excluded the Schorfheide-Chorin from our analyses. Standardised field plots were located in the Schwäbische Alb and in the Hainich-Dün, both middle mountain ranges in Germany. The Schwäbische Alb has an annual mean temperature of 6-7 °C and an annual precipitation of 700-1,000 mm. The geologic parent material is calcareous bedrock of the Oxfordian Age (Epoch: Upper Jurassic) from which Leptosols and Cambisols have developed. The Hainich-Dün has an annual mean temperature of 6.5-8 °C and an annual precipitation of 500-800 mm. The geologic parent material in the Hainich-Dün is calcareous bedrock of the Middle Triassic Epoch partly covered by Pleistocene loess from which Cambisols, Stagnosols, Vertisols and Luvisols have developed.
Location	Biodiversity experiment: The field site is located near the German city of Jena (50°55′ N, 11°35′ E; 130 m above sea level). Agricultural grasslands: The Schwäbische Alb is located between 460-860 m above sea level, the Hainich-Dün is located between 258-550 m above sea level. The exact locations (longitude, latitude) of all 100 plots under study can be found at www.biodiversity-exploratories.de.
Access & import/export	For the biodiversity experiment and the agricultural grasslands, consortium-internal code of conducts had to be signed by each researcher and accordingly, we followed these rules to minimize the environmental impact of our field work. Furthermore, in the biodiversity experiment and in the agricultural grasslands, access to so-called "core areas" was regulated. Finally, digital "field books" kept track of the number of people doing field work and guaranteed restricted access to sensitive plots. Fieldwork permits were issued from 2008 to 2021 by the responsible state environmental offices of Baden-Württemberg (Regierungspräsidium Tübingen), Thüringen (Thüringer Landesverwaltungsamt) and Brandenburg (Landesumweltamt Brandenburg).
Disturbance	We kept the disturbance associated with our study to a minimum by organising joint sampling campaigns (instead of repeated sampling by the different groups involved). The sampling procedure was adjusted in a way to account for the trade-off between representative sampling (large/numerous samples) and the destructive character (e.g., small sampling areas, small soil corers).

Reporting for specific materials, systems and methods

We require information from authors about some types of materials, experimental systems and methods used in many studies. Here, indicate whether each material, system or method listed is relevant to your study. If you are not sure if a list item applies to your research, read the appropriate section before selecting a response.

Materials & experimental systems

n/a	Involved in the study	
[x]	[ ]	Antibodies
[x]	[ ]	Eukaryotic cell lines
[x]	[ ]	Palaeontology and archaeology
[x]	[ ]	Animals and other organisms
[x]	[ ]	Human research participants
[x]	[ ]	Clinical data
[x]	[ ]	Dual use research of concern

Methods

n/a	Involved in the study	
[x]	[ ]	ChIP-seq
[x]	[ ]	Flow cytometry
[x]	[ ]	MRI-based neuroimaging
